# Optical sensor reveals the hidden influence of cell dissociation on adhesion measurements

**DOI:** 10.1038/s41598-024-61485-6

**Published:** 2024-05-22

**Authors:** Kinga Dóra Kovács, Zoltán Szittner, Beatrix Magyaródi, Beatrix Péter, Bálint Szabó, Alexa Vörös, Nicolett Kanyó, Inna Székács, Robert Horvath

**Affiliations:** 1 Nanobiosensorics Laboratory, MFA, Centre for Energy Research, HUN-REN, Budapest, Hungary; 2 Department of Biological Physics, ELTE Eötvös University, Budapest, Hungary; 3 Chemical Engineering and Material Sciences Doctoral School, University of Pannonia, Veszprém, Hungary; 4 Cellsorter Kft., Budapest, Hungary

**Keywords:** Biophysics, Optics and photonics, Biomaterials

## Abstract

Cell adhesion experiments are important in tissue engineering and for testing new biologically active surfaces, prostheses, and medical devices. Additionally, the initial state of adhesion (referred to as nascent adhesion) plays a key role and is currently being intensively researched. A critical step in handling all adherent cell types is their dissociation from their substrates for further processing. Various cell dissociation methods and reagents are used in most tissue culture laboratories (here, cell dissociation from the culture surface, cell harvesting, and cell detachment are used interchangeably). Typically, the dissociated cells are re-adhered for specific measurements or applications. However, the impact of the choice of dissociation method on cell adhesion in subsequent measurements, especially when comparing the adhesivity of various surfaces, is not well clarified. In this study, we demonstrate that the application of a label-free optical sensor can precisely quantify the effect of cell dissociation methods on cell adhesivity, both at the single-cell and population levels. The optical measurements allow for high-resolution monitoring of cellular adhesion without interfering with the physiological state of the cells. We found that the choice of reagent significantly alters cell adhesion on various surfaces. Our results clearly demonstrate that biological conclusions about cellular adhesion when comparing various surfaces are highly dependent on the employed dissociation method. Neglecting the choice of cellular dissociation can lead to misleading conclusions when evaluating cell adhesion data from various sources and comparing the adhesivity of two different surfaces (i.e., determining which surface is more or less adhesive).

## Introduction

The significance of studying cell adhesion has become essential in various fields, including cell biology, materials science, and surface chemistry. The study of cell adhesion can provide insights into fundamental principles of cell biology, such as the role of cell-surface interactions in cell differentiation and tissue development. By understanding how cells adhere to different materials, it becomes possible to develop new biologically active surfaces and medical devices^1,2^ that can interact with cells in specific ways. These advancements are applied in the design of novel materials with both cell-repellent and cell-adhesive properties. Additionally, a thorough characterization of cancer cell adhesion can contribute to our understanding of factors that contribute to tumor progression and metastasis.

A large number of cells are adherent and attach to the extracellular matrix components (ECM), which is a complex network of proteins and other molecules that surrounds cells and provides structural support, via integrin receptors. Integrins are expressed as heterodimers, their composition determines their ligand specificity^3,4^. The tripeptide Arg–Gly–Asp (RGD) was identified as the minimal pattern required to trigger cell adhesion^5–7^. Integrins form different types of adhesion complexes which are linked to the intracellular actin-cytoskeleton system through various adaptor molecules and thus enable mechanotransduction^8^. Mechanotransduction plays a crucial role in regulating cellular functions, differentiation, and proliferation^9–11^. In addition to integrins, cells can also use other adhesion molecules, such as cadherins or selectins, to adhere to other cells or the ECM. These molecules are involved in a range of biological processes, such as tissue development, immune response, and wound healing.

Cell dissociation methods and reagents are widely used in tissue culture laboratories for handling adherent cell types, but their impact on subsequent measurements or tissue engineering is poorly understood. Cell dissociation can have unintended effects on cell behavior, such as altering cell surface markers or inducing stress responses, which can impact the accuracy and reproducibility of downstream experiments.

In standard tissue culture-treated polystyrene dishes cell adhesion is facilitated by the treatment of the bare polystyrene surface with various, mainly oxidizing methods to introduce most importantly hydroxyl and carbonyl groups, and rendering the surface more hydrophilic, and supporting cell adhesion^12^. Upon initial attachment cells produce various ECM proteins, for example, fibronectin and laminin, and thus enable cell adhesion in tissue culture dishes with integrins and form adhesion complexes^13^.

Carrying out experiments with adherent cells requires their dissociation from the bottom of culture dishes. How various cell detachment strategies affect phenotypes of adherent cells is an important issue in cell biology. Mechanical-, enzymatic- and chelation-based cell dissociation approaches are all known for their ability to alter viability, receptor and gene expression, moreover cell response to various stimuli, by disrupting the status quo in adherent cells. This process is unavoidable since the detachment of cells is necessary for experimentation and biomedical applications as well.

Integrins have multiple binding sites for divalent cations and require these ions to take their active and conformation enabling effective ligand–integrin interactions^14^. Chelating these ions through the addition of chelating agents such as EDTA and EGTA renders the integrins, necessary for adhesion, inactive and thus promotes cell dissociation from the substrate. While in some cases EDTA alone is enough to remove cells from the surface, in many cases it is not effective enough and many cells remain attached, resulting in a lower yield. In these cases, more effective methods are required. Moreover, in the case of mesenchymal stem cells, (MSCs) using EDTA also results in lower viability, while retaining the higher chemotactic activity of the cells when compared to enzyme-based methods^15,16^.

The application of proteolytic enzymes, such as trypsin, results in the dissociation of the cells from the surface they adhered to. Proteolytic enzymes act by digesting extracellular proteins, and components of the ECM, the adhesion complexes, and partially the glycocalyx^17^. The superiority of trypsin treatment over the mechanical methods seems established^18^ moreover scraping results in lower viability while increasing the amount of necrotic and apoptotic cells^19,20^.The regularly used 0.05% trypsin for 2 min protocol for cell dissociation does not lead to complete removal of the glycocalyx^21^. However, the density of the glycocalyx seems to contribute to the fine morphology of the plasma membrane^22^. The efficiency of trypsin to cleave certain membrane proteins is difficult to predict even based on the number of trypsin cleavage sites present in each protein, some remain unaffected, while others are almost completely removed in the case of MSCs after 5 min of incubation with the enzyme^23^. Mutein et al. looked at the expression of endothelial markers and found that based on flow cytometry data 1.5 min of incubation with the trypsin–EDTA treatment did not lead to significant changes in the expression of five markers^24^. In general, it is suggested that the expression of each cell surface antigen is to be tested specifically, and the detachment method is to be fine-tuned accordingly^25^. Trypsinization can also influence further biological processes through the proteolysis of the cell surface proteome. This is for example illustrated by the inability of trypsinized tumor cell lysates to induce dendritic cell (DC) maturation compared to tumor cells detached by EDTA only^26^. The intracellular trafficking of integrins allows cells to monitor their environment’s adhesive properties, therefore, it is tempting to speculate that mild trypsinization does not lead to impaired integrin-mediated cellular functions^27^. However, when Doaga et al. compared trypsin–EDTA with only EDTA-treated NIH-3T3 mouse fibroblasts, they found that the adhesion of the non-trypsin-treated cells is faster to collagen I-based scaffolds^28^. Another more direct assay applies single-cell force spectrometry to determine the adhesive properties of cells detached by using either EDTA or trypsin treatment. They found that HeLa cell adhesion properties are not affected by trypsin treatment, unlike in the case of mouse embryonic kidney fibroblasts, and that each cell type is to be assessed for its post-detachment adhesive properties^29^. Trypsin treatment of cells also resulted in alterations in their electrophoretic mobility. Importantly, it was shown that trypsin treatment of HeLa and HL-60 cells affected their electrophoretic mobility (EPM) and the net surface charge can both increase and decrease^30^.

We demonstrate that the application of a label-free surface sensitive optical sensor can precisely quantify the effect of cell dissociation methods on the single cell^31^ and population level^32–34^ cellular adhesivity. Importantly, one can potentially reveal subpopulations with different behaviors even if the population mean is the same. The shape of the single-cell adhesion distribution can also give us additional information^35^. These detailed experiments can be essential in cancer research, where the individual tumor cells can have highly different behaviors^36,37^. The monitoring of cellular adhesion with the high resolution provided by the optical measurements allowed us to quantify the effect of most typical cell dissociation methods on cell adhesion without interfering with the physiological state of the cells. Our findings highlight the importance of carefully considering cell dissociation methods when conducting downstream experiments or tissue engineering and demonstrate the potential of using optical sensors to improve our understanding of cell behavior. In the present study, we demonstrate that the dissociation reagents have a short-term effect on cell adhesion and its population distribution. The experiments involved the use of enzymatic (trypsin–EDTA mixture) and non-enzymatic (EDTA and commercially available dissociation buffer, which contains salts, chelating agents, and cell-conditioning agents in calcium-free and magnesium-free phosphate-buffered saline) dissociation reagents on HeLa cancer cells. Three different surfaces (non-coated (biocompatible metal-oxide), fibronectin-coated, and RGD-coated) were used to study whether the observed effects of dissociation protocols on cell adhesion depend on the adhesion motifs available to the cells. Our results are noteworthy as they demonstrate that when comparing the adhesivity of two different surfaces, such as determining which surface is more or less adhesive, the outcome depends on the cell dissociation method employed.

## Materials and methods

All chemicals and reagents were obtained from Sigma–Aldrich Chemie GmbH (Schelldorf, Germany) unless stated otherwise.

### Cell culture

HeLa cells (93021013 Sigma–Aldrich) were cultured in a humidified incubator at 37 °C with 5% CO_2_ in 60 mm tissue culture petri dishes in Dulbecco’s modified Eagle’s medium (DMEM) supplemented with 100 μg/ml streptomycin and 100 U/ml penicillin mixture(Merck, Germany), 10% fetal bovine serum (Biowest SAS, France) and 4 mM l-glutamine (Merck, Germany).

### Preparation of surface coatings

1 mg fibronectin (human plasma, 354008 Corning) was suspended in 1 ml sterile water and was allowed to go into solution at room temperature for 30 min. The solution was diluted with Dulbecco’s phosphate-buffered saline (DPBS) (pH 7.4) to 31 μg/ml. Each well was coated by adding 10 μl of fibronectin solution. The plate was centrifuged for 10 s at 130×*g* and then incubated at room temperature for 1 h. The remaining material was aspirated, and each well was rinsed with sterile water three times (40–50 μl per well). The plate was air-dried in a hood overnight at room temperature. It was then sealed with aluminum foil and stored at 4 °C. For the experiment, it was used within 2 weeks from the preparation date.

The polymer powders were dissolved in 10 mM 2-[4-(2-hydroxyethyl)piperazin-1-yl]ethanesulfonic acid (HEPES) at 1 mg/ml concentration and stored at − 20 °C. PP (poly(l-lysine)-*graft*-poly(ethylene glycol) (PLL-*g*-PEG, [PLL(20)-g(3.5)-PEG(2)]) (SZ42-28, SuSoS AG, Dübendorf, Switzerland)) and PPR (PLL-*g*-PEG-DBCO-Mal)-CKK-(Acp)-(Acp)-(Acp)- GRGDS (hereafter PP-DBCO-R), obtained as powders from SuSoS AG, Dübendorf, Switzerland (SZ43-74)), were mixed in 1:1 ratio and diluted with 10 mM HEPES to 0.5 mg/ml concentration. 30 μl of this solution was added to each well and the plate was shaken gently for 30 min at room temperature. The solution was aspirated, then the wells were rinsed twice with 40 μl sterile water and filled with the assay buffer (20 mM 2-[4-(2-hydroxyethyl)piperazin-1-yl]ethanesulfonic acid (HEPES) in Hank’s balanced salt solution (HBSS), pH 7.4).

### Resonant waveguide grating optical sensor with single-cell resolution

The Epic Cardio (Corning Incorporated, Corning, NY, USA) is a high-resolution and high-throughput resonant waveguide grating (RWG)-based label-free single-cell resolution optical sensor. For our experiments, Corning Epic 384 well cell assay microplates were used. The bottom of each well has a 2 × 2 mm^2^ RWG sensor area which consists of 80 × 80 pixels with the pixel size of 25x × 25 μm. The microplate is illuminated from below with a tunable broadband light source (wavelength between 825 and 840 nm) and the grating areas couple the light into the thin high refractive index biocompatible Nb_2_O_5_, waveguide layer, which is located on a glass substrate. The coupled light propagates in the waveguide layer with a series of total reflections and then reflects onto a CCD camera. The propagating light creates an evanescent electromagnetic field that decays exponentially with distance from the sensor surface and can interact with the sample placed on the waveguide surface. The instrument detects the wavelength shift of the reflected light with a sensitivity of 0.25 pm within 15,000 pm via the CCD camera with a microplate read time of 3 s.

### Cell dissociation protocols

**Figure 1:**
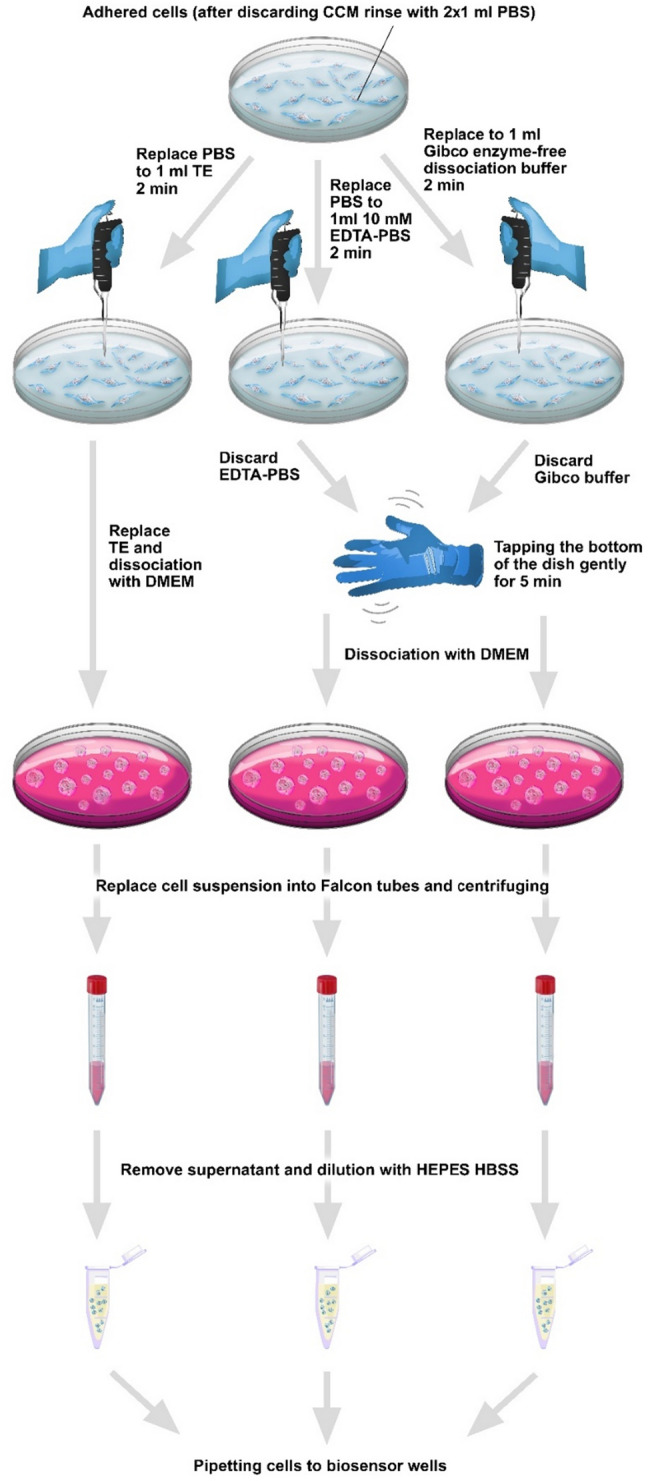
Schematic illustration of the different cell dissociation protocols. First, the cells were treated with different cell dissociation buffers and then shaken if needed. Next, the cells were dissociated from the bottom of the cell culture dish with cell culture media and centrifuged twice to remove the media. Finally, the cells were brought into suspension and diluted with the assay buffer.

The media, reagents, and buffers were warmed up to 37 °C. Adhered HeLa cells in the cell culture dish were rinsed twice with DPBS. After that, the cells were detached from the surface by adding 1 ml of (a) 0.05% trypsin, 0.02% EDTA mixture (Merck, Germany) and incubating for 2 min at room temperature, then dissociation reagent was removed, (b) Gibco’s enzyme-free cell dissociation buffer (13151014 Gibco), incubating for 2 min at room temperature then discarding the buffer and tapping gently the bottom of the dish for 5 min with the lid on, (c) 10 mM EDTA in DPBS, incubating for 2 min at room temperature then discarding the buffer and tapping gently the bottom of the dish for 5 min with the lid on. The reaction of dissociation agents was terminated by adding 1 ml of completed culture medium, cells were collected from the surface of the culture dish, transferred into a 15 ml Falcon tube, and then centrifuged for 5 min at 200×*g*. The supernatant was discarded and cells were resuspended in the 3 ml of assay buffer. The cells were centrifuged again for 5 min at 200×*g*, the supernatant was discarded, and the cells were resuspended in the assay buffer. After cell counting the cell suspension was diluted to the final concentration of 100 cells/25 μl and added into the wells of the sensor plate for the measurement (Fig. 1).

### Cell adhesion assay

**Figure 2:**
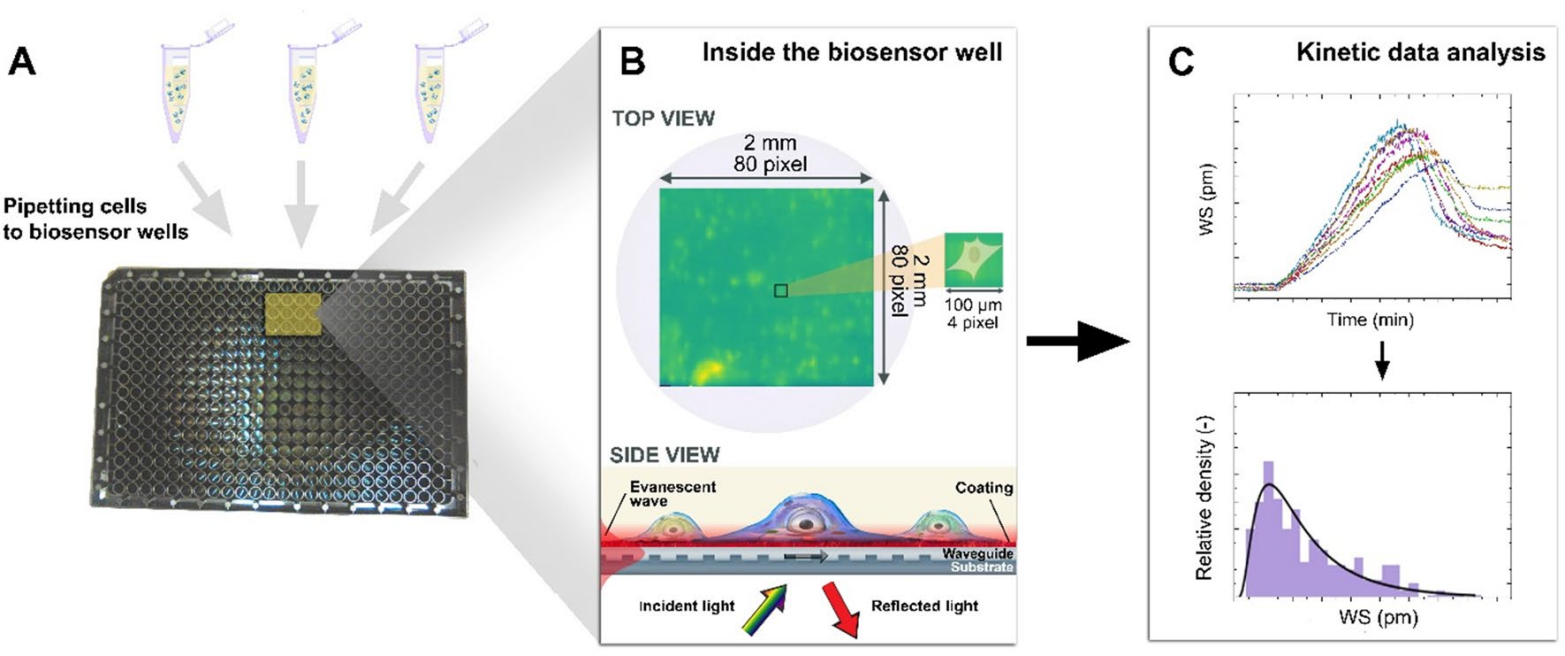
Schematic illustration of the measurement with single-cell resolution optical sensor. The cells were added to 12 wells in the microplate and measured (**A**). The sensor can detect refractive index change above the surface in an approximately 150 nm thick layer, which corresponds to the evanescent field [(**B**), ‘side view’ part, red zone]. The integrin-ligand binding happens in this layer, thus the sensor can monitor cell adhesion and provides kinetic data of the process (**C**).

After the surface coating procedure, 25 μl of assay buffer was used in each well for the baseline in the Epic Cardio sensor. Then the cell suspension was pipetted into the wells, and they were let to fully adhere to the sensor surface for 90 min (Fig. 2A, B). After the sensor signal, measured as the wavelength shift (WS) of the pixel corresponding to the maximal signal of the cell, reached a stable level (see Fig. 2C), the microplate was removed from the instrument and the morphology of the cells was examined with optical microscopy.

### Statistical analysis

Kruskal–Wallis H-test with Wilcoxon post-hoc test was used to analyze the difference between the cell dissociation methods and cell adhesion surfaces because our data doesn’t show normal distribution.

## Results and discussion

The adhesion signal of HeLa cells was measured on three different surfaces (50% PPR, fibronectin, and non-coated) with three different cell dissociation methods (Gibco, EDTA, TE). The sensor data were analyzed as previously described in Sztilkovics et al. Briefly, each cell occupied 1–4 pixels on the sensor surface, and we used the pixel which corresponded to the maximal wavelength shift in our analysis^31^.

The HeLa cell line was selected as a representative cell line for this study due to its widespread use in research as a human cell line, providing insights into numerous fundamental biological processes. Fibronectin is a large extracellular matrix glycoprotein that contains the RGD sequence, which serves as a recognition site for integrins. The RGD-binding integrins comprise α5β1, α8β1, αvβ1, αvβ3, αvβ5, αvβ6, αvβ8, and αIIbβ3^3^. HeLa cells express α5β1, αvβ3, and αvβ5 integrins. These integrins recognize different regions of fibronectin, including the RGD motif. The interaction between integrins and fibronectin is not solely dependent on the RGD sequence; other regions as so-called synergy site Pro–His–Ser–Arg–Asn (PHSRN) of fibronectin also contribute to the binding. The affinity and specificity of integrin binding to fibronectin can vary. The response of other cell types would depend on their specific integrin repertoire and the adhesive surfaces available. Trypsin cleaves peptide bonds at the C-terminal side of lysine and arginine residues. The reaction of trypsin on different types of integrins can vary.

**Figure 3:**
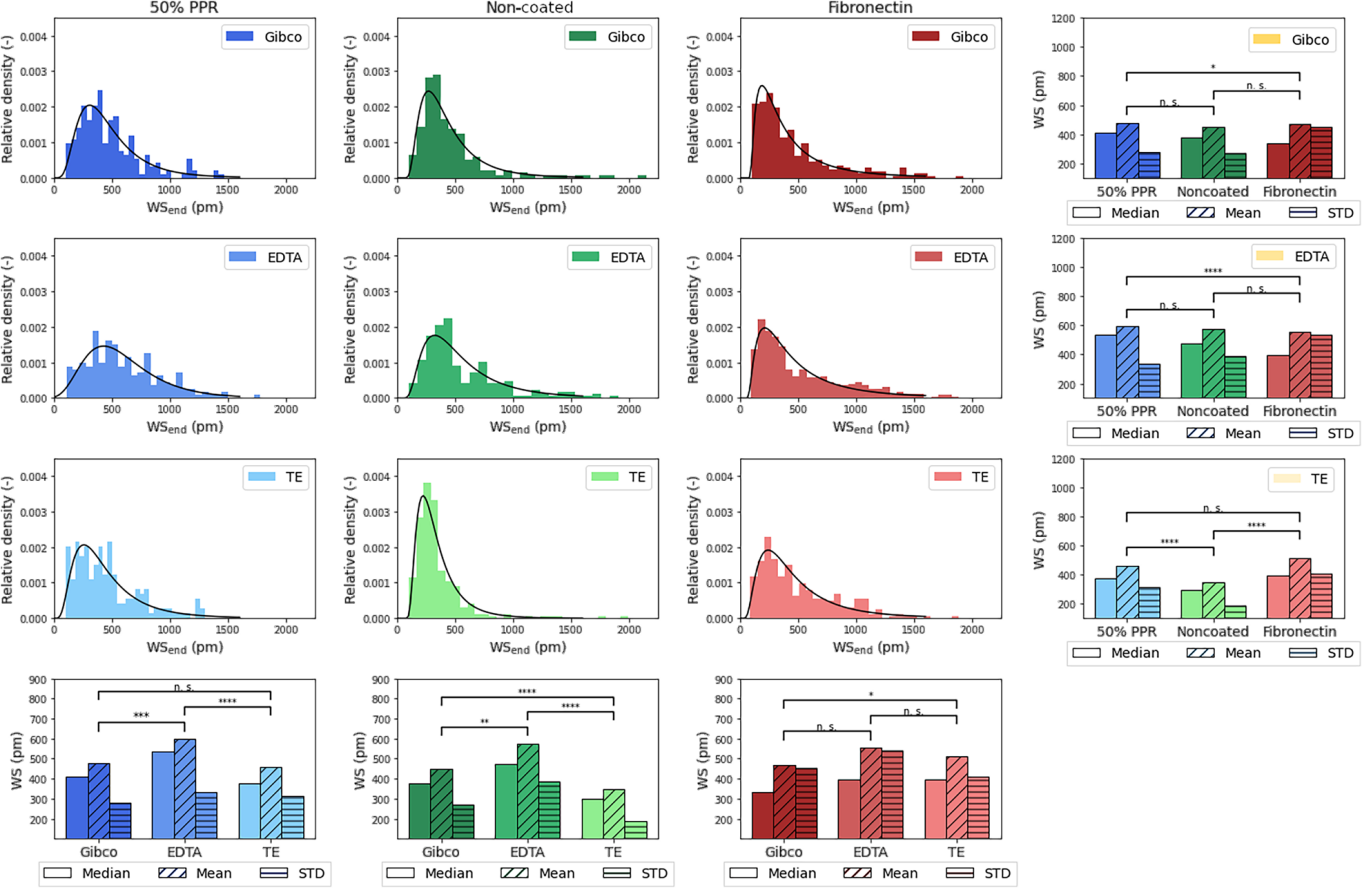
The adhesion signal distribution of HeLa fitted with lognormal distributions, on different surfaces (columns) and with different cell dissociation methods (rows). In the last row the median, mean, and standard deviation of the distributions of the different dissociation methods are depicted. In the last column the median, mean, and standard deviation of the distributions of the different cell adhesion surfaces are depicted. The significance analysis was carried out with a non-parametric Kruskal–Wallis H-test test with Wilcoxon signed-rank test. *p < 0.05, **p < 0.01, ***p < 0.001, ****p < 0.0001.

To compare the single-cell adhesion signals, we plotted the distribution of the last data point of the adhesion signal measured as wavelength shift (WS_end_). Each distribution consists of between 185 and 380 single-cell signals from at least two independent measurements. We tested the reproducibility of the experiments and found that there is no significant difference in the adhesion signals distribution with a correlation coefficient of 0.965. A lognormal distribution was obtained for all surfaces with every dissociation method, as shown in Fig. 3. Non-parametric Kruskal–Wallis H-test test with Wilcoxon signed-rank test was used to quantitatively compare the effect of the different dissociation methods on the different surfaces. The largest effect of the different cell dissociation methods was observed on the non-coated surface, whereas there was barely a difference on the fibronectin-coated surface.

To determine the effect, we compared the distribution of WS_end_, a measurement of cell adhesion strength, of single cells harvested using different cell dissociation reagents on various surfaces (Fig. 3 and Table 1).

**Table 1:**
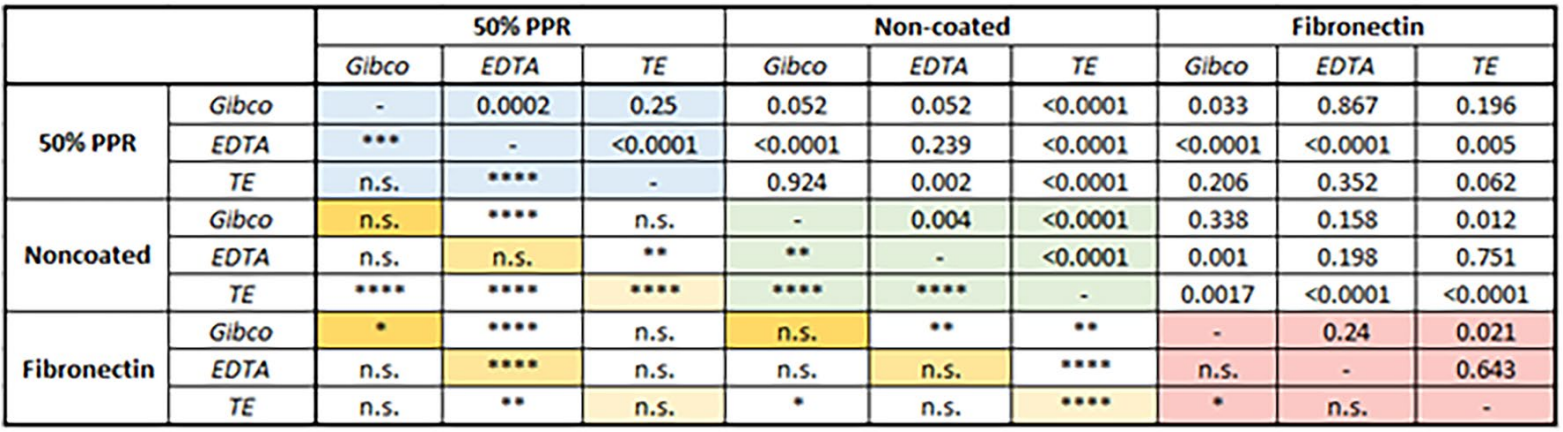
The p values from the non-parametric significance analysis.

With blue, green, and red backgrounds we depicted the p values plotted in the last row of Fig. 3. With yellow background we depicted the p values plotted in the last column of Fig. 3.

*p < 0.05, **p < 0.01, ***p < 0.001, ****p < 0.0001.

Firstly, we compared the distribution of cell adhesion signals obtained by collecting the cells using different dissociation reagents on the same surface. On fibronectin-coated surfaces, only the Gibco-TE comparison showed a significant difference. Interestingly, this comparison showed no difference on the 50% PPR surface. However, both the Gibco-EDTA and EDTA–TE comparisons showed significant differences in the distribution of cell adhesion signals. Surprisingly, all comparisons resulted in significant differences in the cell adhesion distribution on the non-coated surface. The application of TE resulted in a more uniform cell adhesion distribution profile, leading to highly significant differences when compared to the enzyme-free EDTA and Gibco-based methods. Altogether, these results suggest that the more biologically relevant the surface studied for cell adhesion, the less the cell adhesion is affected by employing another dissociation method.

Next, the WS_end_ distribution of single cells obtained by various cell harvesting methods was compared on different surfaces. Both Gibco and EDTA reagents showed similar effects, with both only showing significant differences in the comparison of fibronectin and 50% PPR. However, in the case of TE, these comparisons yielded inverted results, and significant differences were only found when comparing cell adhesion on non-coated surfaces with both 50% PPR and fibronectin surfaces.

These results underscore the significant impact of different cell dissociation reagents on cell adhesion across different surfaces. To further demonstrate the importance of cell dissociation in the process of cell adhesion, we also compared the distribution of WS_end_ signals across all conditions. For example, the comparison of cell adhesion on non-coated and 50% PPR-coated surfaces did not result in significant differences when cells were harvested using EDTA. However, replacing EDTA with either Gibco or TE for cells applied on the non-coated surface showed highly significant differences in the distribution of cell adhesion signals for the same comparison. Similarly, the comparison of the 50% PPR surface with the fibronectin surface, when both cell populations were harvested using EDTA, showed highly significant differences. However, if the cells on the 50% PPR surface were collected using Gibco or TE, the differences were abolished and non-significant.

Our findings highlight the contribution of the cell harvesting method to cell adhesion, especially in the early and nascent phases of the process, when adhesion complexes have a submicron size (~ 0.1 μm in diameter)^38^ dot-like^39^ structure that exists only for 2–10 min, and consists of about 50 integrins with a key role in the attachment process of cells to the ECM. Furthermore, our results suggest that the measured differences on various surfaces are highly dependent on the cell harvesting method used. These considerations highlight the importance of careful interpretation of previously published data. Especially since not only TE and EDTA-based harvesting modified the cell adhesion but also the additives present in commercial cell dissociation reagents. Next to changes in receptor expression and net surface charge of the cell, viability likely also plays a role in the process of early cell adhesion investigated in this study.

This difference in the cell adhesion distribution between the surfaces can be due to the different biological ‘activeness’, where on the fibronectin-coated surface, cells can find various cell adhesion motifs readily available so the extent of the digestion of the surface proteins does not matter a lot. On the 50% PPR-coated surface, where only RGD cell adhesion motifs are available to the cells, for optimal adhesion the cells need their integrin receptor, glycocalyx, and surface proteins intact^21^. Therefore, the extent of the digestion of these elements from the cell surface will influence the cell adhesion, the weakest adhesion was observable with the enzyme-treated cells (Fig. 3). We also compared the effect of glycocalyx digestion with ChrABC enzyme^35^ to the effect of the different cell dissociation methods. Importantly, enzyme addition resulted in a wider distribution, similarly to the effects of EDTA and GIBCO compared to TE (see Fig. S1). However, enzyme addition at large concentration resulted in a weakly adhering subpopulation completely missing in case of cell dissociation treatment, and interestingly the changes in the median and mean were opposite. Therefore, more work is needed to in depth understand the molecular scale changes caused by the various dissociation protocols, presumably being a complex process influencing many cell adhesion factors in a complicated manner. On the non-coated surface there are no cell adhesion motifs, the cells adhere to surface through a passive process, so digestion which effect the cell’s charge greatly influences the cell adhesion.

**Figure 4:**
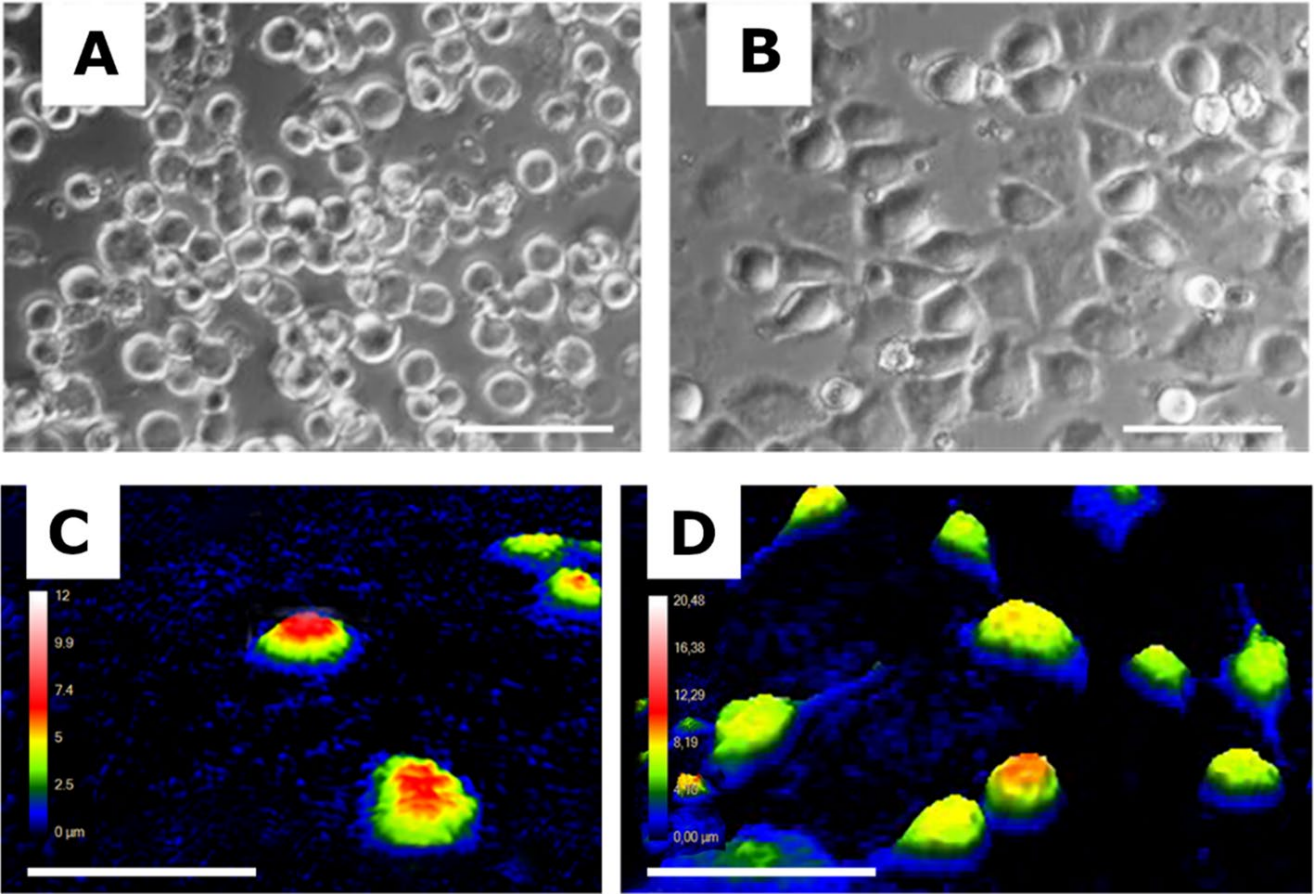
Microscopic image of HeLa cells on (**A**) non-coated and (**B**) 50% PPR-coated sensor surface. Holomonitor images of HeLa cells on (**C**) non-coated and (**D**) 50% PPR-coated sensor surface. For all images TE was used as the dissociation protocol. On the non-coated surface, there are no cell adhesion motifs, the cells adhere to the surface through a passive process, so the cells have a hemispherical shape.

In the images shown in Fig. 4, it is evident that cells on the non-coated surface exhibit a hemisphere-like shape, indicating passive adhesion. However, moving to a more biologically active substratum, the 50% PPR-coated surface, the cells appear to be more spread out and exhibit more irregular shapes.

**Figure 5:**
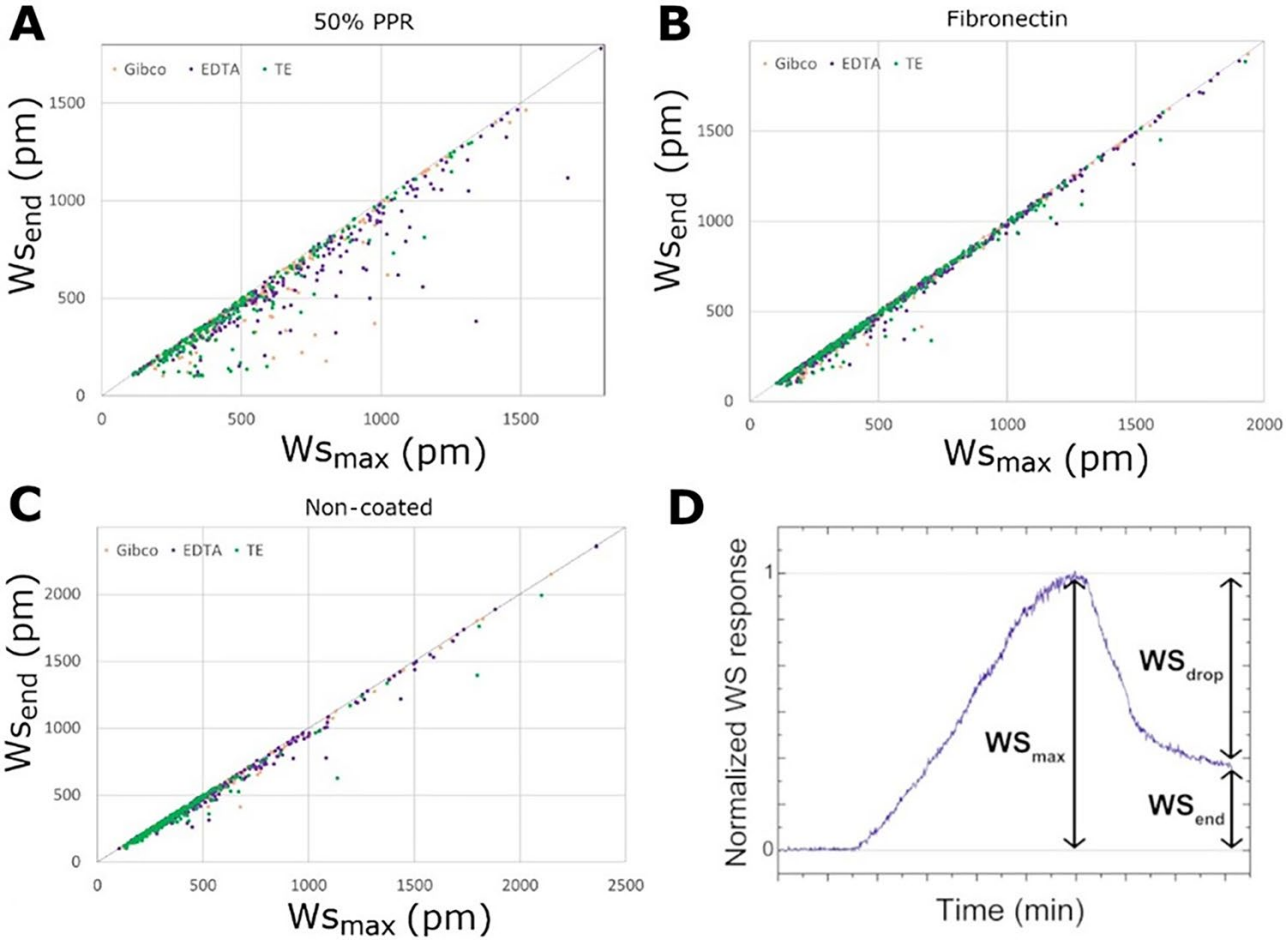
(**A**–**C**) Correlation scatterplots show the last wavelength shift values plotted (WS_end_) as a function of the maximal wavelength shift (WS_max_) for each measured cell, detached with the various dissociation methods (orange, purple, green) on the three different surfaces. (**D**) Normalized (with WS_max_) single-cell kinetic data with a drop in the wavelength signal.

As shown in Fig. 5D, a signal drop was sometimes observed in the single-cell kinetic data, which usually follows a simple sigmoidal curve. We compared this phenomenon between different surfaces and cell dissociation methods and plotted the ratio of the maximum of the curve (WS_max_) and the last data point (WS_end_) for each individual cell. The results are shown in Fig. 5. Clearly, there is no drop in the single-cell adhesion signal if the dot representing the given cell is on the y = x line. We found that this phenomenon was only notable on the 50% PPR-treated surface regardless of the dissociation methods.

To further analyze this phenomenon, we plotted the distributions of the extent of the signal drop for each cell dissociation method on all surfaces (Fig. S2). Subsequently, we quantified the size of the subpopulation exhibiting a notable signal drop (1−WSend /WSmax > 0.1 or 0.2), as presented in Table S1 and Table S2. We noticed that the size of the drop depended greatly on the type of surface but not the dissociation method.

Of note, in the employed coatings, the averaged RGD-RGD density is predefined, around 8.63 nm, and the RGD motifs deposited are largely localized^21^. This is in contrast to other types of coatings where freely diffusing RGD motifs are deposited using thiol groups on gold^40,41^. Therefore, this relatively rigid surface structure of RGD motifs can potentially lead to suboptimal local RGD–RGD distances, leading to adhesion weakening, through creating mature adhesion contacts with decreased stability. It is still interesting why this phenomenon only happens for a distinct subpopulation of cells (see SI Table S1, S2). On the non-coated surface, there is a strong passive adhesion, independent of adhesion contacts, and the surface is also completely homogeneous, therefore the cells do not have the opportunity or the driving force for any kind of movement governed by biologically active motifs on the surface. We believe, the signal drop phenomenon cannot be attributed to increased motility, as evidenced by the literature demonstrating that cell motility is lower on the PPR-coated surface compared to the fibronectin-coated surface^42^. Additionally, the presence of the PHSRN synergy peptide in the fibronectin molecules promotes cell motility and migration, aligning with the aforementioned statement^43,44^. We believe, however, more work should be done to test the above hypotheses and reveal the exact nature of the subpopulation showing relatively fast adhesion signal drops after reaching the adhesion plateau.

Due to the fact, that the cell adhesion signals are clearly dependent on not only the surface but also on the above-investigated cell dissociation methods, it is important to consider other dissociation methods to eliminate the observed effects from the adhesion measurements. The most recent reagent-free cell detachment methods, such as acoustic waves^45,46^ and temperature-responsive cell culture dishes^47,48^, have the potential to enable cell detachment with minimal impact on the cells. However, the effects of these approaches have yet to be compared in detail to classical methods.

## Conclusions

We demonstrated that the various cell dissociation methods (Gibco, EDTA, TE) have a significant impact on cell adhesion, and this effect is also influenced by the surface (non-coated biocompatible metal-oxide, fibronectin-coated, and RGD-coated) used in the adhesion experiments. The most pronounced effect of the dissociation methods was observed on the non-coated metal-oxide surface, while only a slight difference was discernible on the fibronectin-coated surface. Our measurements were conducted using a high-throughput label-free optical sensor, allowing us to eliminate the influence of labeling and enabling the measurement of adhesion signals at the single-cell level with statistics. Consequently, we were able to in-depth analyze the population distribution of cell adhesion signals on multiple surfaces using different cell dissociation methods. Our findings emphasize the importance of using enzyme- and chelating-free cell dissociation techniques in adhesion measurements. Additionally, it is crucial to consider the impact of different cell dissociation methods during the validation of new biologically active surfaces and other biomaterials, especially when comparing the adhesivity of various surfaces. Conclusions stating that one surface is more adhesive than the other can be misleading or even incorrect if the choice of cell dissociation method is overlooked. Moreover, the platform used to study cellular adhesion kinetics could potentially open up novel application directions in antibody screening^49^, monitoring environmental contaminants^50^ and antibody-based biosensors^51–53^ due to its excellent lateral and temporal resolution and flexibility in available surface coatings.

### Supplementary Information

Supplementary Information.

## Data Availability

The datasets used and/or analyzed during the current study available from the corresponding author on reasonable request.
