# Supplementary material for: Optical sensor reveals the hidden influence of cell dissociation on adhesion measurements
Source: Sci Rep. 2024 May 22;14:11719. doi: 10.1038/s41598-024-61485-6 (PMC11111754; doi:10.1038/s41598-024-61485-6)
Supplement: Supplementary file 1 — Supplementary Information. [file 41598_2024_61485_MOESM1_ESM.docx]

**Supplementary Information**

**Optical Sensor Reveals the Hidden Influence of Cell Dissociation on Adhesion Measurements**

*Kinga Dóra Kovács^1,2^, Zoltán Szittner^1^, Beatrix Magyaródi^1^, Beatrix Péter^1^, Bálint Szabó^2,3^, Alexa Vörös^1^, Nicolett Kanyó^1^, Inna Székács^1^, Robert Horvath^1,*^*

^1^ *Nanobiosensorics Laboratory, MFA, Centre for Energy Research, HUN-REN, Budapest, Hungary*

*^2^ Department of Biological Physics, Eötvös University, Budapest, Hungary*

*^3^ Cellsorter Kft., Budapest, Hungary*

**e-mail:* [*horvath.robert@ek.hun-ren.hu*](mailto:horvath.robert@ek.hun-ren.hu)

*
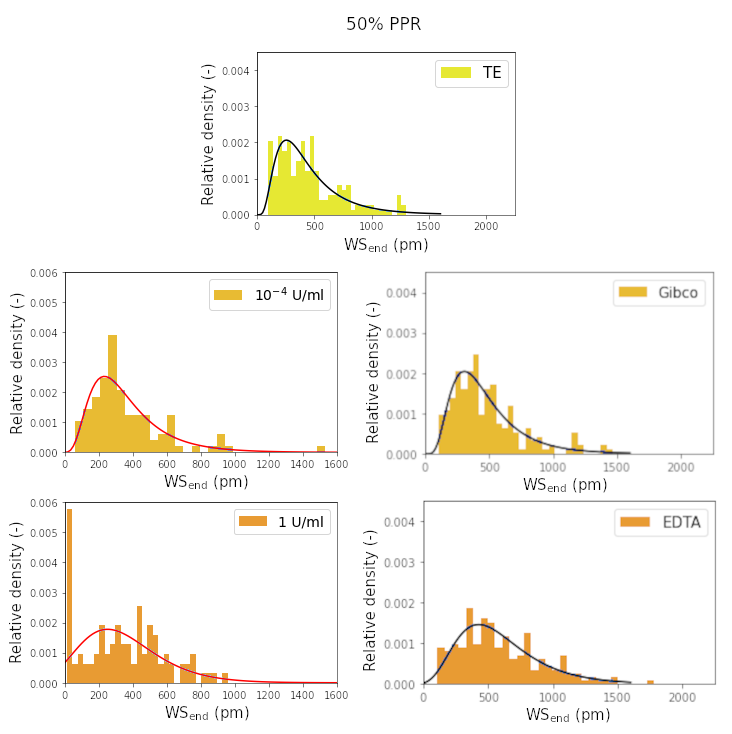
*

**Figure S1.** *The adhesion signal distribution of HeLa fitted with lognormal distributions on 50% PPR-coated surface with glycocalyx digestion (ChrABC enzyme) and TE cell dissociation (first column) and with different cell dissociation methods (second column). The median and mean exhibit a monotonic decrease, while the standard deviation increases with higher enzyme concentration compared to lower enzyme concentration, resulting in a wider distribution. For Gibco and EDTA compared to TE, the effect on the distributions' median and mean are the opposite; they show an increase instead of a decrease. However, the impact on the standard deviation remains the same, the Gibco distribution has a smaller standard deviation than that of the EDTA distribution.* *(It is important to note that TE was used in the glycocalyx digestion experiments.)*

| ***> 0.1*** | **50 % PPR** | **Non-coated** | **Fibronectin** |
| --- | --- | --- | --- |
| **Gibco** | 31% | 11% | 11% |
| **EDTA** | 45% | 15% | 13% |
| **TE** | 39% | 21% | 15% |

**Table S1.** *The ratio of cells with a signal drop larger than 0.1 (1-WS_end_ /WS_max_ > 0.1) for the three cell dissociation methods on the three different surfaces.*

| ***> 0.2*** | **50 % PPR** | **Non-coated** | **Fibronectin** |
| --- | --- | --- | --- |
| **Gibco** | 14% | 1% | 4% |
| **EDTA** | 18% | 3% | 4% |
| **TE** | 18% | 3% | 5% |

**Table S2.** *The ratio of cells with a signal drop larger than 0.2 (1-WS_end_ /WS_max_ > 0.2) for the three cell dissociation methods on the three different surfaces.*


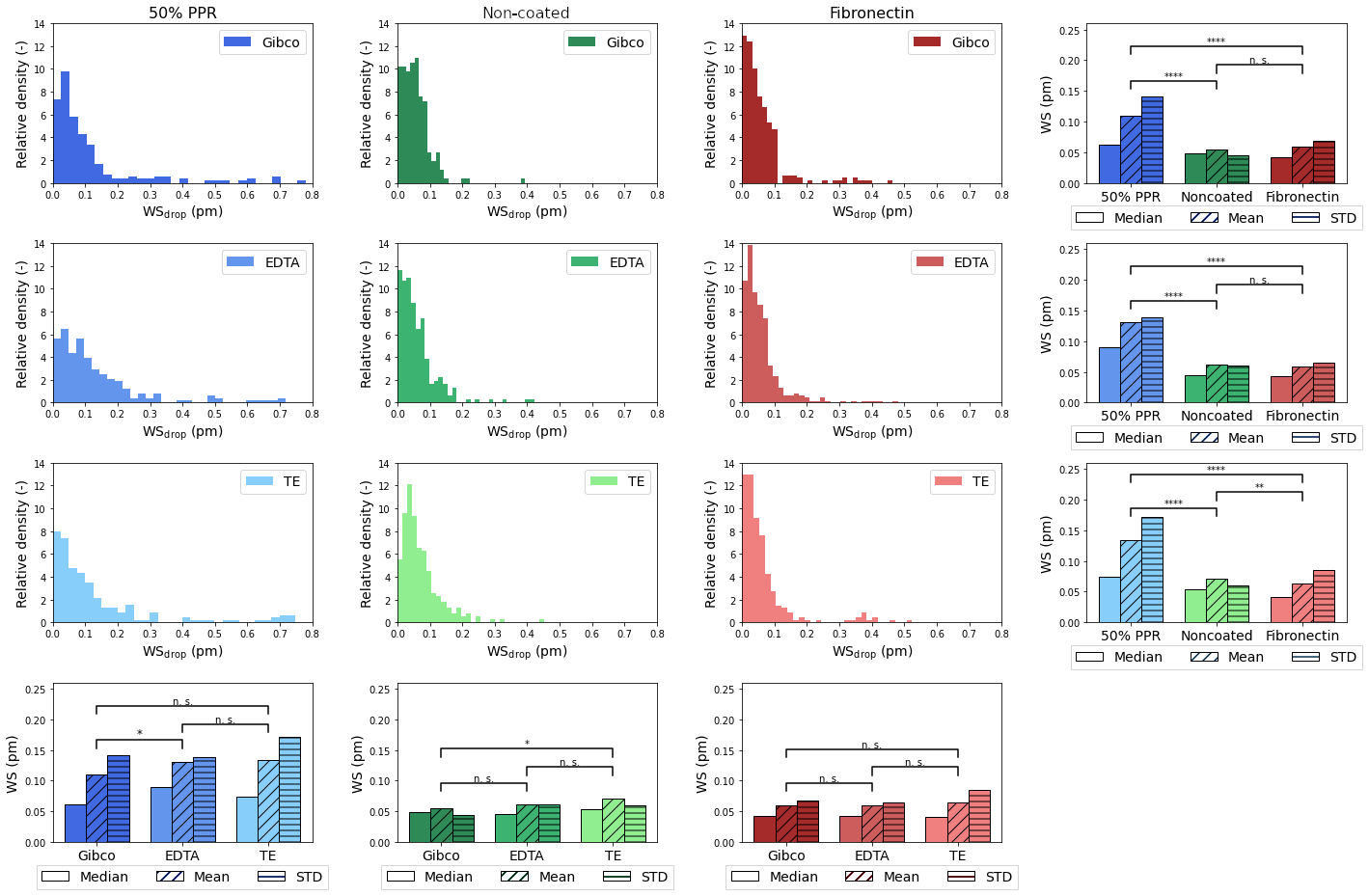


**Figure S2.** *The sensor signal drop distribution of HeLa, on different surfaces (columns) and with different cell dissociation methods (rows). In the last row, the distributions of the different dissociation methods' median, mean, and standard deviation are depicted. In the last column, the median, mean, and standard deviation of the distributions of the different cell adhesion surfaces are shown. The significance analysis was carried out with a non-parametric Kruskal-Wallis H-test test with Wilcoxon signed-rank test. p<0.05: *, p<0.01: **, p<0.001: ***, p<0.0001: *****
